# Supporting Mental Health Among STEM Students: The REDFLAGS Model

**DOI:** 10.3390/bs15111559

**Published:** 2025-11-14

**Authors:** Michael T. Kalkbrenner, Noelle A. Filoteo Young

**Affiliations:** Department of Counseling and Educational Psychology, New Mexico State University, Las Cruces, NM 88003, USA; nfiloteo@nmsu.edu

**Keywords:** The REDFLAGS Model, student well-being, STEM, mental health, university training

## Abstract

While the future of college student mental health is leaning towards systemic-level integrated behavioral health care models, existing mental health support for science, technology, engineering, and mathematics (STEM) students remains highly individual. The REDFLAGS Model is a mental health resource comprising an acronym of warning signs that suggest a college student might be struggling with mental distress. The aim of this study was to test the utility of The REDFLAGS Model, with a large sample of STEM students (*N* = 358). Results revealed support for the latent dimensionality of The REDFLAGS Model among a large sample of STEM students. Results also demonstrated that higher recognition of the items on The REDFLAGS Model as warning signs for mental distress was a significant predictor of peer-to-peer referrals to counseling among STEM students. Additionally, STEM students with help-seeking histories and those who identified as female were more likely to recognize the items on The REDFLAGS Model as warning signs of mental distress than those without help-seeking histories and men, respectively. Collectively, results indicated that The REDFLAGS Model has potential to provide college counselors with an empirically supported framework for supporting STEM student mental health. It is available at no cost and can be shared in print or digital formats.

## 1. Introduction

### 1.1. Supporting Mental Health Among STEM Students: The REDFLAGS Model

Students pursuing degrees in science, technology, engineering, and mathematics (STEM) fields represent a distinctive college population that faces unique mental health challenges (Daker et al., 2021; Kalkbrenner et al., 2022; Pester et al., 2023). Compared to students in non-STEM fields, those in STEM disciplines are often less equipped at identifying warning signs of mental distress, and they are less likely to seek out mental health services. This is particularly concerning, as the demanding and highly competitive nature of STEM programs can further contribute to the increased risk of psychological distress among these students (Jensen et al., 2023; Posselt & Lipson, 2016). A number of academic and financial resources exist to support STEM students (Leoni et al., 2023; National Science Foundation, n.d.; Rennar-Potacco et al., 2016; Ulappa et al., 2022). However, the extant literature is lacking research on supporting STEM students’ mental health, which is an essential factor for student retention, success, and well-being both inside and outside of the classroom (Chu et al., 2022; LeViness, 2024; Thomas et al., 2021). To these ends, counselors and other university staff who work with STEM students need mental health support resources with utility for distribution on both microsystemic and macrosystemic levels.

**Figure 1:**
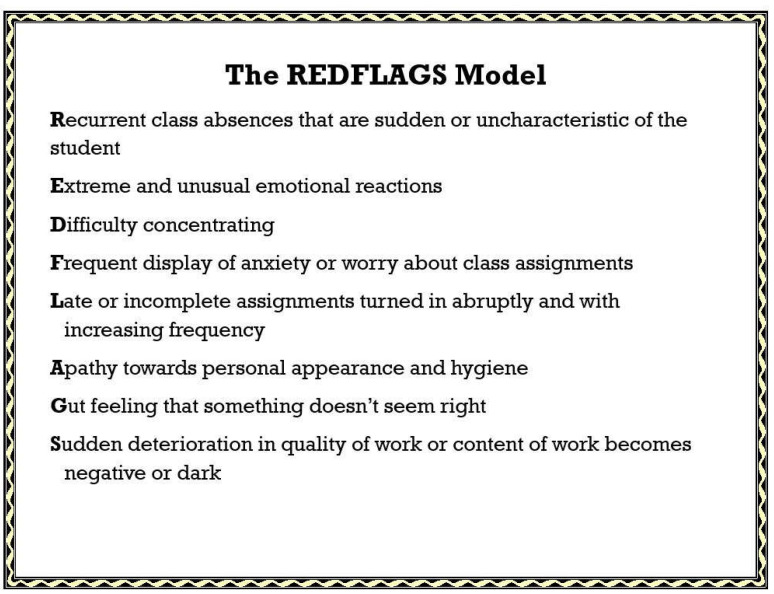
The REDFLAGS Model. From “Recognizing and supporting students with mental disorders: The REDFLAGS Model,” by M. T. Kalkbrenner, 2016, *Journal of Education and Training*, *3*(1), p. 5. © Kalkbrenner (2016). Reproduced with permission.

The REDFLAGS Model (see Figure 1) is a mental health resource comprising an acronym of warning signs that suggest a college student might be struggling with mental distress (Kalkbrenner, 2016). The REDFLAGS Questionnaire is a standardized screening tool for measuring the extent to which test takers recognize the items on the model as warning signs for mental distress (Kalkbrenner et al., 2020a). To date, past investigators (e.g., Kalkbrenner & Carlisle, 2021; Kalkbrenner et al., 2020a) found empirical support for the latent dimensionality of The REDFLAGS Model with four different college-based populations. Despite the growing evidence in favor of the generalizability and utility of The REDFLAGS Model with different college-based populations, the model has not been tested with STEM students. Counselors and their constituents have a responsibility to ensure that standardized tests (e.g., The REDFLAGS Questionnaire) and the theoretical models they appraise (The REDFLAGS Model) are validated with untested populations before using them in practice or for research purposes (Lenz et al., 2022). If validated with STEM students, The REDFLAGS Model has potential to offer college counselors a tool for supporting STEM student mental health.

### 1.2. Mental Health Among STEM Students

Research suggests that those in the STEM academic community, including students, suffer from high levels of burnout, depression, and anxiety (Pester et al., 2023). Specific to STEM disciplines, the culture of prioritizing work over self-care needs, reports of bullying and public shaming of students, and anxiety stemming from expected research competency can play a role in exacerbating mental health distress (Henderson, 2023; Limas et al., 2022; Pester et al., 2023). The onus of ensuring mental well-being is placed solely on STEM students, instead of examining common practices and expectations of STEM students that may contribute to mental health distress (Limas et al., 2022). Past investigators found a number of intersectional demographic variables that can exacerbate mental distress among STEM students (Posselt & Lipson, 2016; Wilkins-Yel et al., 2022).

#### Demographic Differences

Membership in particular demographic groups, especially minoritized groups, can exacerbate mental health distress among STEM students (Posselt & Lipson, 2016). According to the National Center for Science and Engineering Statistics (NCSES, 2023) women obtained 50% of all total bachelor’s degrees in STEM. However, underrepresentation is noticeable in some STEM disciplines, including physical and earth sciences, mathematics, computer science, and engineering (NCSES, 2023). Data show that women in STEM are less likely to win funding awards for research, less likely to publish, more likely to get their manuscripts rejected in the peer review process, and less likely to be included as a co-author by men who obtain research grants (Jebsen et al., 2022). The competitive and challenging environment that women STEM students experience can lead to higher levels of stress, depressive symptoms, anxiety symptoms, and endorsement of suicidal ideation in severe cases (Reidy & Wood, 2024; Wilkins-Yel et al., 2022). STEM culture can also lead to lower retention rates for women STEM students (Ortiz-Martínez et al., 2023), which may contribute to the lower percentage of women in STEM occupations compared to men (NCSES, 2023). Moreover, first-generation students tend to receive lower grades in STEM subjects and are also less likely to have parents in STEM careers (Bettencourt et al., 2020). The competition in STEM disciplines may also influence retention and mental health (Canning et al., 2020; Limas et al., 2022). First-generation STEM students are more likely to change majors when compared to non-first-generation students (Fabiano, 2022) and are more likely to screen positive for both anxiety and depression if they perceive a competitive environment (Posselt & Lipson, 2016).

In addition to gender identity and generational status, ethnoracial identity is another important variable that should also be examined when considering STEM students’ mental health. Underrepresentation in STEM disciplines is still evident, particularly for students who identify as Hispanic, Black, or American Indian (NCSES, 2023). Lack of representation can affect one’s sense of belonging (Miles et al., 2020). Further, ethnoracial minorities in STEM fields may be the recipients of microaggressions, whether verbal or behavioral in nature (Lee et al., 2020; Miles et al., 2020). These microaggressions can invalidate one’s identity as a STEM student and tend to be related to poorer mental health outcomes (Miles et al., 2020). In addition, help-seeking history is an increasingly investigated variable in the college counseling literature (Cheng et al., 2018). Kalkbrenner et al. (2021) found that college students with help-seeking histories (sought one or more sessions of counseling) were more likely to recognize the items on REDFLAGS Model as warning signs for mental distress when compared to those without help-seeking histories. It is important to consider the intersectionality of STEM students’ different identities and social locations, which can heighten mental distress in STEM fields.

### 1.3. Ecological Systems Approaches: College Student Mental Health

Students in need of mental health care services in a college or university may come across microsystemic and macrosystemic barriers that prevent them from obtaining professional help (Shea et al., 2019). Students may not be aware of available services on their respective campuses (Cronin et al., 2021). Students may also want to avoid social stigma attached to help-seeking behavior, such as being perceived as academically incompetent, feelings of embarrassment, or being shunned from social circles (Radez et al., 2021; Wada et al., 2019). One way that college counselors and their constituents have worked to address these issues in recent years, is by shifting towards ecological systems level approaches for supporting students’ health and wellness (Woodruff & Boyer, 2024).

As Bronfenbrenner (1992) explains in his ecological systems theory, human development is shaped by numerous macro-and-micro-level systems. An individual may be most directly affected by more direct concerns (microsystem), but these concerns are influenced by cultural, social, and economic factors that cannot be directly controlled (macrosystem). An expansion of the original model by Bronfenbrenner and Morris (1998) discussed the Process-Person-Context-Time (PPCT) model, where all four elements interplay and affect overall human development. Although originally formulated for younger children, research has shown that the PPCT model can be adapted at any stage of the lifespan, including for individuals in higher education (Renn & Smith, 2023), particularly for context where changes in environment and peer groups influence physical and mental health outcomes for college students. These types of holistic approaches to college student health and wellness oftentimes include integrated behavioral health models in which medical and mental health professionals work together to support students’ combined mental and physical health (Readdean et al., 2024). Despite this trend, mental health care for STEM students tends to be highly individual and lacks ecological systemic supports (Limas et al., 2022).

#### Peer-to-Peer Mental Health Support

Peer-to-peer mental health support is a systems approach to wellness, which uses the power of shared understanding and experiences to provide both practical and emotional support to peers in similar situations (Smit et al., 2022). Research has shown that students tend to turn to their peers before family members or figures of authority when experiencing a mental health crisis (Born This Way Foundation, 2022; Dimitropoulos et al., 2024). Studies have shown positive effects of peer support, providing increased levels of happiness and coping skills and reduced levels of stress, depression and anxiety (Pointon-Haas et al., 2023; Richard et al., 2022). However, even with the recognition of mental health symptoms in peers, students may be reluctant to provide support due to lower levels of confidence (Kim & Hong, 2021).

Providing the necessary skills training to college students for peer-to-peer support may help foster social connection and decrease stigmatization surrounding mental health treatment, while also increasing knowledge about referral options when professional help is needed (Caporale-Berkowitz, 2020). Past investigators found that The REDFLAGS Model was a resource for promoting peer-to-peer mental health support with a number of college-based populations (Kalkbrenner & Carlisle, 2021; Kalkbrenner et al., 2020a). If validated with STEM students, the model might have utility for supporting peer-to-peer support among STEM students.

### 1.4. The REDFLAGS Model

The REDFLAGS Model is an acronym of possible signs of mental distress ranging from observable behavior to quality of academic work (Kalkbrenner, 2016; see Figure 1). The “R” stands for recurrent class absences, while the “E” refers to extreme or unusual emotional reactions. The “D” in the acronym represents difficulty concentrating, while the “F” pinpoints frequent anxiety or worry about assignments. The “L” in the acronym stands for late or incomplete assignments, while the “A” discusses apathy or a lack of care towards personal appearance and hygiene. The letter “G” stands for one’s gut feeling or intuition about something not being right, and the “S” talks about sudden deterioration in the quality of work a student is submitting (Kalkbrenner, 2016). The findings of a number of past investigations demonstrated support for the latent dimensionality of The REDFLAGS Model with a number of college-based populations, for example, four-year university students (Kalkbrenner et al., 2020a), faculty members (Kalkbrenner & Carlisle, 2021), and first-generation community college students (Kalkbrenner et al., 2021). In each of these past investigations, participants’ ability to recognize the items on The REDFLAGS Model as warning signs for mental distress emerged as a statistically significant predictor of making one or more referrals to the college counseling center.

### 1.5. The Present Study

The validity of screening tools and theoretical frameworks may differ across populations. It is the ethical duty of counselors to confirm that standardized assessments and theoretical models are supported by validated data before applying them in clinical practice or research (Lenz et al., 2022). At the time of this writing, the internal structure of The REDFLAGS Model has not been tested with STEM students. Accordingly, the primary aims of the present study were to first test the latent dimensionality of The REDFLAGS Model with STEM students. If STEM students’ scores on the REDFLAGS Model are validated, we will proceed to test the capacity of the model for predicting peer-referrals to counseling as well as investigate demographic differences in STEM students’ recognition of the items on the model as warning signs for mental distress.

The results of this study have potential to offer college counselors an empirically supported mental health awareness model that is free to use and distribute. In addition, results might provide initial support for the utility of peer-to-peer support among STEM students as well as identify groups of STEM students who are more and/or less aware of warning signs of mental distress. College counselors might use this information to direct mental health support outreach on campus. To these ends, the following research questions (RQs) guided the present investigation: RQ1. Is the latent dimensionality of the REDFLAGS Questionnaire confirmed with STEM students? RQ2. Are STEM students’ scores on the REDFLAGS Questionnaire predictive of making at least one peer-to-peer referral to the counseling center? RQ.3 Are there demographic differences in STEM students’ ability to recognize the items on the REDFLAGS Model as warning signs of mental distress?

## 2. Methods

A quantitative cross-sectional research design was used to answer the research questions. A subjects-to-variables ratio (STV) of >10:1 or at least 200 participants and a priori power analysis (Faul et al., 2007) were used to determine the minimum necessary sample size for factor analysis based on a structural equation model. The REDFLAGS Questionnaire (see Section 2.3) is a brief screening tool comprising eight items. Thus, a sample size of at least 200 was necessary for factor analysis. For the logistic regression and factorial analysis of variance (ANOVA), the results of a power analysis showed that a sample size of at least 249 would provide an 80% power estimate, alpha = .05, with a moderate effect size, f = .25.

### 2.1. Procedures

Following IRB approval, we acquired a list of email addresses from the Office of University Student Records, comprising undergraduate students enrolled in STEM majors at a research-intensive university with four campuses in three cities in the Southwestern United States. Data collection spanned multiple campuses in various cities to secure an adequate sample size for statistical analysis and to enhance the data’s representativeness. A recruitment message was then distributed to these students through the Qualtrics Secure Online Survey Platform. 407 prospective participants clicked on the electronic link.

A response rate could not be determined, as Qualtrics does not monitor inactive email addresses. 37 cases were excluded due to completely missing data. In all likelihood, these prospective participants clicked on the link and then decided not to participate. Among the remaining data *N* = 370, z-scores showed 10 univariate outliers and Mahalanobis distances revealed two multivariate outliers, which were removed, yielding a final sample of *N* = 358 undergraduate STEM students. All skewness and kurtosis values were less than an absolute value of 1, indicating the data were highly consistent with a normal distribution.

This study is part of a larger project with an aim to validate scores on health-based screeners and promote wellness among STEM students. In the present study, we further analyzed the data set in Kalkbrenner and Miceli (2022) to answer completely different research questions. Specifically, Kalkbrenner and Miceli (2022) investigated the psychometric properties of a barriers to counseling scale among STEM students. There are a few differences in the demographic profiles between the current study (see Section 2.2) and Kalkbrenner and Miceli (2022) due to the differences in the research questions between the separate studies. More specifically, these differences were due to differences in missing values and outliers, as the current study analyzed different variables than Kalkbrenner and Miceli (2022). For example, there were 41 missing cases in Kalkbrenner and Miceli (2022); however, there were 37 missing cases in the present study. This difference was due to the use of different variables between this study and Kalkbrenner and Miceli (2022). In other words, more participants completed the instrumentation for the variables we tested in this study than the variables that were tested by Kalkbrenner and Miceli (2022).

### 2.2. Participants

Participants (*N* = 358) ranged in age from 18 to 63 (*M* = 24.70; *SD* = 8.70). For gender identity, 65.6% (*n* = 235) self-identified as female, 29.9% (*n* = 107) male, 2% (*n* = 7) non-binary, 1.1% (*n* = 4) transgender, .6% (*n* = 2) reported that they were still figuring out their gender identities, and .8% (*n* = 3) preferred not to specify their gender identities. In terms of ethnoracial identity, 49.4% (*n* = 177) of participant self-identified as Hispanic, Latinx, or of Spanish origin, 36.3% (*n* = 130) White or European American, 3.6% (*n* = 13) multiracial, 3.1% (*n* = 11) Asian American, 2.5% (*n* = 9) American Indian or Alaska Native, 2.2% (*n* = 8) Black or African American, 1.4% (*n* = 5) Middle Eastern or North African, .8% (*n* = 3) another race, ethnicity, or origin, and .6% (*n* = 2) that preferred not to specify their ethnoracial identity. In terms of generational status, 63.4% (*n* = 227) of participants indicated that they were non-first-generation college students, while 36.6% (*n* = 131) indicated that they were first-generation college students. Finally, 53.1% (*n* = 190) of participants denied any help-seeking history, while 46.4% (*n* = 166) indicated help-seeking history, and .6% (*n* = 2) of responses to this question were missing.

### 2.3. Instrumentation

Participants read an informed consent statement, confirmed that they met the inclusion criteria (18 years old or older, presently enrolled in at least one undergraduate STEM course, and currently a STEM major), responded to a demographic questionnaire, and then completed the REDFLAGS Questionnaire. The demographic questionnaire solicited information regarding participants’ gender identities, ethnoracial identities, help-seeking histories, and generational status. Participants also specified their age in years and if they had referred one or more peers to the counseling center.

#### REDFLAGS Questionnaire

The REDFLAGS Questionnaire measures the extent participants endorse items on The REDFLAGS Model as possible signs of mental distress (Kalkbrenner et al., 2020a). Participants respond to items using a 5-point Likert-type scale with the following anchor definitions: 1 (I strongly disagree that this behavior is a sign of a mental health issue.), 2 (I disagree that this behavior is a sign of a mental health issue.), 3 (I’m not sure if this behavior is a sign of a mental health issue.), 4 (I agree that this behavior is a sign of a mental health issue.), and 5. (I strongly agree that this behavior is a sign of a mental health issue.). Mean scores are computed for the eight items, with higher scores denoting a greater ability to recognize signs of mental distress (Kalkbrenner et al., 2020a).

Previous researchers established the latent dimensionality of The REDFLAGS Model and questionnaire through both exploratory factor analysis and confirmatory factor analyses (CFAs; Kalkbrenner & Carlisle, 2021; Kalkbrenner et al., 2020a, 2020b, 2021). Past investigators also found strong evidence for the internal consistency reliability of scores on The REDFLAGS Questionnaire, ranging from α = .89 (Kalkbrenner et al., 2020a) for undergraduate college students, to α = .90 (Kalkbrenner & Carlisle, 2021) with faculty members, and α = .91 (Kalkbrenner et al., 2021) to α = .92 (Kalkbrenner et al., 2020b) with community college students. Similarly, acceptable internal consistency reliability estimates of scores on The REDFLAGS Questionnaire emerged with the present sample of STEM students (α = .832 [95% CI = .801, .858], ω = .834, [95% CI = .799, .860]).

### 2.4. Data Analyses

A CFA with a maximum likelihood estimation method was computed in IBM SPSS AMOS version 29 to test the latent dimensionality of STEM students’ scores on the REDFLAGS Questionnaire (RQ1). We followed the following combined guidelines of Dimitrov (2012) and Schreiber et al. (2006) for interpreting model fit in CFA: Chi square absolute fit index (*CMIN*, non-significant *p*-value or χ^2^ to *df* ≤ 3), the normative fit index (*NFI*, .90 to .95 = acceptable fit and ≥ .95 = strong fit), the comparative fit index (*CFI*, .90 to .95 = acceptable fit and ≥ .95 = strong fit), the standardized root mean square residual (SRMR < .08), and the root mean square error of approximation (*RMSEA* ≤ .08 with 90% *CIs*). Assessing internal consistency and reliability of scores is a crucial precursor to testing for latent dimensionality, as test scores cannot be valid without being reliable. While Cronbach’s alpha (α) is the most widely used estimate of internal consistency, its accuracy depends on the data meeting specific statistical assumptions (McNeish, 2018). Composite reliability estimates, such as McDonald’s omega (ω), often provide more stable reliability measures. Therefore, both α and ω with 95% confidence intervals (*CIs*) were calculated in this study.

We performed a logistic regression analysis to explore whether STEM students’ scores on the REDFLAGS Questionnaire could predict one or more peer referrals to the counseling center (RQ2). The predictor variable was students’ interval-level composite score on the REDFLAGS Questionnaire. The criterion variable was measured on a categorical scale. As part of the demographic questionnaire, students answered the following question: “Have you ever referred (recommended) another student to counseling services?” and selected either “0 = *never referred a peer to the counseling center*” or “1 = *referred one or more peers to the counseling center*.”

A factorial analysis of variance (ANOVA) was computed to answer the third research question regarding demographic differences in STEM students’ recognition of the REDFLAGS items as warning signs for mental distress. The four categorical level independent variables and corresponding levels included, gender identity (female or male), ethnoracial identity (Latinx, White, or other), help-seeking history (yes or no), and generational status (first-generation college student or non-first-generation college student). The levels of the gender and ethnoracial identity variables were coded to create comparison groups that were large enough for statistical analyses. This aggregation procedure comes with limitations to external validity; however, this procedure can be appropriate in survey research pending the authors are transparent about its limitations (Ross et al., 2020). The limitations of the coding procedure will be explained in Section 4. The results of a Levene’s test showed that the assumption of homogeneity of error variances had not been violated, *F*(23, 316) = .744, *p* = .799.

## 3. Results

The REDFLAGS Questionnaire items were entered into a CFA and with the exception of the *CMIN*, (χ^2^ [20] = 63.60, *p* < .001, χ^2^ to *df* = 3.18), a satisfactory model fit emerged: *CFI* = .95; *NFI* = .93; *RMSEA* = .08, 90% *CI* (.06, .10); and the *SRMR* = .04. The standardized factor loadings (*M* = .63; *SD* = .09) were acceptable-to-strong and ranged from .44 to .74. Considering that the *CMIN* tends to underestimate model fit with large samples (Dimitrov, 2012), we proceeded with inferential statistical testing (i.e., answering RQs 2 and 3), as the collective CFA results confirmed the latent dimensionality of STEM students’ scores on the REDFLAGS Questionnaire. In other words, the CFA results revealed that The REDFLAGS Questionnaire was appropriately calibrated for answering RQs 2 and 3.

A logistic regression analysis was conducted to answer RQ2 on the predictive capacity of STEM students’ scores on the REDFLAGS Questionnaire. STEM students’ composite score on The REDFLAGS Questionnaire were entered into the model as the predictor variable. The categorical level criterion variable was if participants have made at least one peer-to-peer referral to the counseling center (0 = never referred a student to the counseling center or 1 = has referred a student to the counseling center). The logistic regression model was statistically significant, *X*^2^ (1) = 85.37, *p* < .001, Nagelkerke *R*^2^ = .052. The odds ratio, *Exp(B)*, demonstrated that an increase of one unit in students’ recognition of the items on The REDFLAGS Model as warning signs for mental distress was associated with an increase in the odds of having made at least one peer-to-peer referral to the counseling center by a factor of 2.21.

A factorial 2(gender) X 3(ethnicity) X 2(help-seeking history) X 2(generational status) ANOVA was computed to investigate demographic differences in STEM students’ recognition of the REDFLAGS items as warning signs for mental distress (RQ3). A statistically significant main effect emerged for gender identity, *F* = (1, 316) = 11.19, *p* = .001, $\eta_{p}^{2}$ = .034. Female STEM students scored higher (*M* = 4.20) on the REDFLAGS Questionnaire than male students (*M* = 3.84). A statistically significant main effect also emerged for help-seeking history, *F =* (1, 316) = 5.21, *p* = .023, $\eta_{p}^{2}$ = .016. STEM students with a help-seeking history scored higher (*M* = 4.11) on the REDFLAGS Questionnaire than STEM students without a help-seeking history (*M* = 3.89).

## 4. Discussion

The aims of the present study were to (a) test the latent dimensionality of The REDFLAGS Model with a normative sample of STEM students, (b) test the capacity of STEM students’ recognition of the warning signs on the model for predicting one or more peer-referrals to counseling, and (c) examine demographic differences in STEM students’ recognition of the content of the model as warning signs for mental distress. We will discuss the results in accordance with the RQs.

The results of the CFA revealed satisfactory support for the latent dimensionality of The REDFLAGS Model with STEM students (RQ1). This finding suggests that The REDFLAGS Model and its dimensions were satisfactorily estimated with a normative sample of STEM students. Collectively, the CFA results are consistent with and advance the extant literature in support of the utility of The REDFLAGS Model. Specifically, the dimensionality of The REDFLAGS Model has been confirmed with the following normative samples: four-year university students (Kalkbrenner et al., 2020a), faculty members (Kalkbrenner & Carlisle, 2021), community college students (Kalkbrenner et al., 2020b) and first-generation community college students (Kalkbrenner et al., 2021). The CFA findings in this study extend this evidence to a sixth normative sample of STEM students. The CFA in the present study was based on structural equation modeling, which makes the results indicative of a confirmation of a theoretical model as well as a psychometric test (Mvududu & Sink, 2013). The CFA results in the present study, coupled with past investigations of the dimensionality of The REDFLAGS Model (Kalkbrenner & Carlisle, 2021; Kalkbrenner et al., 2021) suggest that the items on the model comprise a unidimensional theoretical model of warning signs for mental distress.

The results of the LR revealed that higher scores on the REDFLAGS Questionnaire were a statistically significant predictor of STEM students having made one or more peer-referrals to the counseling center (RQ2). This finding is consistent with past investigations of The REDFLAGS Model with different college-based populations (e.g., Kalkbrenner & Carlisle, 2021; Kalkbrenner et al., 2019, 2021). Collectively, the findings of the present study and past investigations suggest that college students and faculty members with higher levels of awareness of the warning signs for mental distress on The REDFLAGS Model tend to report higher odds of having referred one or more students to the counseling center. Perhaps students and faculty members who are more familiar with how to recognize warning signs of mental distress in their peers or students are more comfortable making a referral to the counseling center. Future inquiry is needed to investigate this possible implication of the LR results.

The factorial ANOVA revealed that female STEM students and STEM students with help-seeking histories scored higher on The REDFLAGS Questionnaire than male students and students without help-seeking histories, respectively (RQ3). Similarly, among a general sample of undergraduate students, Kalkbrenner et al. (2020a) found that students who identified as female scored significantly higher on The REDFLAGS Model than male students. Gender roles at both the macro and micro levels can contribute to men’s reluctance to seek counseling (Shepherd et al., 2023). For male STEM students, this reluctance may be even stronger due to the intersection between traditional gender roles and the high-pressure STEM environment, which often discourages vulnerability (Lipson et al., 2016; Rafal et al., 2018). Specifically, the pressure to avoid appearing vulnerable, combined with the competitive academic demands in STEM, may lead male students in these fields to be particularly hesitant to recognize or acknowledge warning signs for mental distress.

Men tend to be less likely than women to acknowledge mental health issues and seek treatment (Shepherd et al., 2023). Consequently, male STEM students may undervalue the importance of counseling and be less inclined than their female peers to view mental distress as a serious health concern. It may also be helpful to contextualize these factors through the lens of the PPCT model (Bronfenbrenner & Morris, 1998). As reported by Renn and Smith (2023), contextual factors such as a competitive academic environment, learned behavior from peers, and gendered social norms play a role in how mental distress is perceived and addressed for college students in STEM. Future research is needed to explore these potential factors further.

Consistent with past investigations with different college based-samples (e.g., Kalkbrenner et al., 2021) found that students with help-seeking histories scored higher on The REDFLAGS Questionnaire than those without help-seeking histories. This finding might indicate that attending counseling may strengthen STEM students’ abilities to recognize warning signs of mental distress in their peers. More specifically, direct experience with counseling may have a psychoeducational component. This finding is especially important, as STEM students represent a distinct college population with unique mental health needs and typically lower rates of mental health service usage than non-STEM students (Daker et al., 2021; Kalkbrenner et al., 2022; Lipson et al., 2016).

### 4.1. Limitations and Future Research

We advise readers to consider the limitations of this study before the implications for practice. The CFA results in this study supported the latent dimensionality of The REDFLAGS Model with a sample of STEM students. However, the non-probability sampling technique is a limitation to the external validity of the findings. Relatedly, it is important to acknowledge that STEM students are not necessarily a uniform group. Future research could explore this further by conducting factorial invariance testing to determine if The REDFLAGS Questionnaire maintains psychometric consistency across different subgroups within STEM fields. For example, future researchers might test the factorial invariance of The REDFLAGS Model (i.e., if the meaning of the model is the same) between students in different science, technology, engineering, or mathematics majors.

The CFA results in the present study are the fifth test of internal structure validity evidence of scores on The REDFLAGS Questionnaire (i.e., sixth college-based sample). Future researchers can extend this line of research by testing for convergent and/or divergent validity evidence of STEM students’ scores on The REDFLAGS Questionnaire with other established mental health screening tools. In terms of the second research question, it is important to note that the results of a logistic regression analysis are based on odds rather than probability. Future researchers could build on this work by conducting pretest/posttest evaluations to assess the extent to which increased awareness of The REDFLAGS Model causes an increase in peer-to-peer referrals to the counseling center. Additionally, future researchers can build on this line of research by investigating how STEM students’ scores on The REDFLAGS Questionnaire might change over time.

The self-report bias is a limitation in survey research. Participants’ responses might consciously (or unconsciously) be influenced to appear more favorable or socially acceptable, reducing the accuracy of the data. This can lead to discrepancies between reported behaviors, attitudes, or experiences and their actual occurrence. Additionally, although our results showed no demographic differences by ethnoracial identity in STEM students’ awareness of the items on the model as warning signs for mental distress, our use of dummy-coding to compare racial/ethnic groups (Latinx, White, or other ethnicity) may have limited the findings. Grouping all non-White and non-Latinx students together may have missed some of the potential variations within this category. Future studies should aim to examine counseling barriers among more racially and ethnically diverse STEM samples. Future researchers might incorporate The REDFLAGS Model in an online app to increase the versatility of distribution.

### 4.2. Implications for Practice

The results of this study have several implications for enhancing the practice of college counselors and other university staff that work with STEM students. In recent years, universities in the United States are adopting systems-level integrated behavioral health models to provide more comprehensive care (Readdean et al., 2024; Woodruff & Boyer, 2024). The results from this study showed that the REDFLAGS Model might have utility for supporting STEM student mental health. Our results indicated that higher scores on the REDFLAGS Questionnaire or recognizing items on the questionnaire as indicative of signs of mental distress were correlated with an increase in the odds that a student made a peer-to-peer referral for professional mental health services. We also found certain demographic characteristics, such as previous help-seeking history and identifying as female, were correlated with higher scores on the REDFLAGS Questionnaire. These results, as well as the extant literature (e.g., Limas et al., 2022; Pester et al., 2023) that discusses mental health among students in STEM fields, have several implications for college counselors and the utilization of The REDFLAGS Model in practice.

Symptoms of mental health distress, such as higher levels of anxiety and depression, have been found in STEM students (Pester et al., 2023). Consistent with finding from the extant literature with different college student populations (e.g., Kalkbrenner et al., 2021), we found that STEM students with help-seeking histories were more likely to endorse items on the REDFLAGS Model as symptoms of mental health distress. The responsibility of mental health care for STEM students has been highly individual in recent years (Limas et al., 2022). One way that college counselors can incorporate systems-level mental health support is through coordinating outreach programs with STEM departments focusing on self-care habits for students, as well as strategies to take care of themselves amidst the demands of their STEM programs. College counselors can use The REDFLAGS Model as a resource during outreach events to increase student capability of recognizing the warning signs on the model as possible symptoms of mental health distress in both themselves and in their peers. Awareness of counseling services available on campus should also be emphasized in these outreach programs, as not seeking professional help may be because of lack of awareness in the student body (Cronin et al., 2021).

The REDFLAGS Model can be distributed in hard copy and/or digital format. College counselors may find The REDFLAGS Model useful in communicating signs of mental health distress in an easily digestible format for those who may have difficulty with more clinical jargon. For example, The REDFLAGS Model has been found to have utility with faculty members, who might be well-positioned to spot these signs in their students (Kalkbrenner & Carlisle, 2021). Moreover, since peers are more likely to discuss mental health distress with other peers rather than faculty (Dimitropoulos et al., 2024), college counselors can use The REDFLAGS Model during outreach events to increase student capability of recognizing warning signs of mental health distress by providing psychoeducation on the connection of items on the REDFLAGS Model with mental health disorders. College counselors can compare pre/post scores on the REDFLAGS Questionnaire as one way to measure the utility of psychoeducation sessions. Universities across United States are shifting towards systems-level integrated behavioral health models (Readdean et al., 2024; Woodruff & Boyer, 2024). Accordingly, there might be utility in posting The REDFLAGS Model in waiting rooms in integrated behavioral healthcare centers on campus.

Informed by the tendency of college students to discuss mental health distress with peers (Born This Way Foundation, 2022), mental health outreach events may also be designed to discuss and combat the stigma associated with seeking mental health support services. College counselors may use extant research about the benefits of seeking professional help for college students (LeViness, 2024) to inform their presentations. For example, college counselors and their constituents can coordinate with the directors of STEM programs to arrange for brief mental health awareness presentations (Williams et al., 2024).

Counselors can increase buy in from directors of STEM programs and STEM faculty by teaching them about the academic and personal benefits that attendance in counseling can have for their students. Some benefits that have been found through workshops conducted with STEM community members (including faculty, staff, and students) included higher levels of confidence in not only showing other people compassion but also exhibiting self-compassion as well (Williams et al., 2024). Further, Williams et al. (2024) also found a higher willingness to discuss mental health concerns with other STEM community members. Programs and workshops increasing community support may help change attitudes that STEM students’ mental health is an individual, and not community, concern (Limas et al., 2022).

Our findings revealed that STEM students with help-seeking histories were more likely to recognize warning signs of mental health distress on the REDFLAGS Questionnaire than those without help-seeking histories. Psychoeducation about the utility of peer-to-peer referrals and making resources more available for students to disseminate with peers may help increase numbers of peer-to-peer referrals. When working with STEM students, college counselors may discuss, when appropriate, how to best provide a referral to a college counseling center and the procedures involved in making a referral. College counselors may also work in conjunction with other university offices, such as the office of the dean of students or university student councils, to create printed or digital copies of The REDFLAGS Model.

### 4.3. Summary and Conclusion

There were three primary aims in the present study, including (a) evaluate the latent dimensionality of The REDFLAGS Model with a normative sample of STEM students, (b) assess how well STEM students’ ability to recognize the model’s warning signs predicted the odds of making one or more peer-referrals to counseling, and (c) explore whether recognition of these warning signs varied across different demographic groups within the STEM student population. Results of a CFA supported the latent dimensionality of STEM students’ scores on The REDFLAGS Questionnaire (standardized screening tool for measuring the extent to which test takers recognize the items on The REDFLAGS model as warning signs for mental distress).

The results of a logistic regression analysis demonstrated that higher scores on The REDFLAGS Questionnaire significantly predicted greater odds of one or more peer-referral to the counseling center among STEM students. Tests of demographic differences showed that female STEM students and STEM students with a help-seeking history were significantly more likely to recognize items on The REDFLAGS model as warning signs for mental distress than male students and those without a help-seeking history, respectively.

Despite the growing integrated-behavioral healthcare models on college campuses (Readdean et al., 2024; Woodruff & Boyer, 2024), mental health care for STEM students tends to be highly individual and systemic-level supports (Limas et al., 2022). The REDFLAGS Model might be a promising tool for supporting STEM student mental health considering the heightened mental health challenges that STEM students often encounter (Henderson, 2023; Limas et al., 2022; Pester et al., 2023) coupled with the need for systems-level STEM student mental health support (Limas et al., 2022). The REDFLAGS Model offers college counselors an empirically supported mental health awareness model, which is free to use and can be distributed via hard copies and/or electronically.

## Data Availability

The original contributions presented in this study are included in the article. Further inquiries can be directed to the corresponding author.
